# Bellmunt Risk Score as a Prognostic Tool in Metastatic Castration-Resistant Prostate Cancer Survival

**DOI:** 10.1001/jamanetworkopen.2026.0300

**Published:** 2026-03-06

**Authors:** Thomas Büttner, Niklas Klümper, Jörg Ellinger, Manuel Ritter, Philipp Krausewitz

**Affiliations:** 1 Department of Urology and Pediatric Urology, University Hospital Bonn, Bonn, Germany

## Abstract

**Importance:**

Metastatic castration-resistant prostate cancer (mCRPC) is an incurable disease with a highly variable clinical course, necessitating robust prognostic tools to guide individualized patient management.

**Objective:**

To validate the prognostic ability of the Bellmunt Risk Score (BRS) for predicting survival in patients with mCRPC.

**Design, Setting, and Participants:**

For this prognostic study, a post hoc analysis was conducted of 2 international, multicenter, phase 3 randomized clinical trials of men (aged ≥18 years) with mCRPC (ACIS [first-line setting] and ELM-PC-5 [postdocetaxel setting]). The ACIS trial enrolled patients between December 2014 and August 2016. The ELM-PC-5 trial enrolled patients between October 2010 and February 2013. Data analyses were conducted between September 2024 and March 2025.

**Exposure:**

Baseline BRS (range, 0-3), calculated by assigning 1 point for each of 3 factors (Eastern Cooperative Oncology Group Performance Status score ≥1, hemoglobin level <10 g/dL, and liver metastases).

**Main Outcomes and Measures:**

The primary outcomes, overall survival (OS) and radiographic progression-free survival, were assessed via Kaplan-Meier and multivariable Cox proportional hazards regression analysis.

**Results:**

This analysis included 678 evaluable male participants from the ACIS trial (median age, 71 years [range, 48-92 years]) and 1078 from the ELM-PC-5 trial (median age, 70 years [range, 43-89 years]), for a total of 1756 participants. The median follow-up was 54.8 months (IQR, 51.5-58.4 months) in the ACIS trial and 10.7 months (IQR, 0.4-27.1 months) in the ELM-PC-5 trial. Having a higher BRS was consistently associated with unfavorable OS in both cohorts. In the ACIS trial, the median OS decreased from 42.2 months (95% CI, 35.7-46.7 months) for patients with a BRS of 0 to 9.1 months (95% CI, 3.7 months to not reached) for those with a BRS of 3. Adjusted hazard ratios (AHRs) for OS were 1.37 (95% CI, 1.12-1.67) for patients with a BRS of 1, 2.64 (95% CI, 1.89-3.69) for a BRS of 2, and 8.29 (95% CI, 2.57-26.78) for a BRS of 3 compared with those with a BRS of 0. In the ELM-PC-5 trial, the median OS decreased from 23.0 months (95% CI, 21.50 months to not reached) for patients with a BRS of 0 to 3.2 months (95% CI, 1.4-8.6 months) for those with a BRS of 3. AHRs for OS were 1.65 (95% CI, 1.31-2.07) for patients with a BRS of 1, 2.93 (95% CI, 2.22-3.87) for those with a BRS of 2, and 4.43 (95% CI, 2.65-7.40) for those with a BRS of 3 compared with those with a BRS of 0. The BRS remained a robust and independent prognostic factor for both outcomes in all multivariable models.

**Conclusions and Relevance:**

In this prognostic study of patients with mCRPC, the BRS was validated as a prognostic tool that provides clinically meaningful information across different treatment lines. Due to its simplicity, the BRS is a practical aid for guiding treatment decisions and facilitating prognostic discussions in routine clinical care.

## Key Points

**Question:**

Is the Bellmunt Risk Score (BRS) a valid prognostic tool for predicting survival in patients with metastatic castration-resistant prostate cancer (mCRPC)?

**Findings:**

In this prognostic study using data for 1756 patients with mCRPC in the ACIS and ELM-PC-5 phase 3 trials, having a higher BRS was consistently associated with unfavorable overall survival and radiographic progression-free survival. The BRS remained a robust and independent prognostic factor for both outcomes in multivariable models.

**Meaning:**

These findings suggest that the BRS provides validated and clinically meaningful prognostic information that can aid in guiding treatment decisions and risk stratification in patients with mCRPC.

## Teaser

This prognostic study uses data from 2 randomized clinical trials to assess whether the Bellmunt Risk Score is a valid tool for predicting survival in patients with metastatic castration-resistant prostate cancer (mCRPC).

## Introduction

Metastatic castration-resistant prostate cancer (mCRPC) is an advanced disease state marked by progression despite androgen deprivation and dissemination to distant sites.^1^ Despite substantial therapeutic advancements in recent years, mCRPC remains an incurable disease with a universally limited prognosis.^1,2^ Notably, the clinical course of patients with mCRPC is highly heterogeneous, with individual survival times varying widely. Both the adverse effects of prior therapeutic interventions and individual patient-specific contextual factors play a decisive role. Critical determinants for therapeutic decision-making include cumulative toxicity from previous treatments, residual physiological resilience, and the overall burden of the current malignant neoplasm.^3,4,5,6,7,8^ The inherent variability in prognosis of mCRPC presents a substantial challenge for both clinicians and patients, making it difficult to accurately predict individual outcomes, guide treatment intensity, stratify patients for clinical trials, and manage patient expectations.^3,4,9,10^ Moreover, substantial interindividual variation exists in how patients weigh anticipated survival benefits against potential adverse effects, highlighting the imperative of prognostic discussions as an integral component of shared decision-making.^11,12^

Robust prognostic tools are essential to guide individualized management in mCRPC, enabling intensified treatment or trial enrollment for patients at high risk while avoiding overtreatment in those at lower risk.^10,13^ Current prognostic models in mCRPC—including mostly single markers such as prostate-specific antigen (PSA), PSA doubling time, alkaline phosphatase, lactate dehydrogenase, or the presence of visceral metastases—often lack the comprehensive predictive power needed to fully capture the complex interplay of factors influencing mCRPC prognosis.^10,14,15,16,17^

The Bellmunt Risk Score (BRS) was initially developed as a prognostic tool for patients with metastatic urothelial carcinoma to predict overall survival (OS) following failure of first-line platinum-based chemotherapy,^18^ using readily available clinical parameters that reflect both patient resilience and tumor burden. The BRS has since become an established baseline characteristic in this indication.^19^ Recognizing the unmet need for available and reliable prognostic instruments in mCRPC, our research group previously identified the BRS as a promising predictor of OS in a pilot mCRPC cohort.^20^ Robust and validated prognostic scores such as the Halabi and Armstrong models incorporate numerous variables (8 and 11, respectively).^21,22^ The BRS, by contrast, relies on just 3 readily accessible, dichotomous variables: Eastern Cooperative Oncology Group Performance Status (ECOG PS) scores,^23^ hemoglobin levels, and presence of liver metastases. This simplicity is the primary strength of the BRS, making it a highly pragmatic tool for rapid risk stratification at the point of care. However, to ensure clinical utility, any prognostic score must demonstrate robust predictive validity in large, independent, and prospectively collected datasets.

Hence, the primary aim of the current study was to validate the prognostic capabilities of the BRS in 2 large, prospective, and well-characterized cohorts of patients with mCRPC: the ACIS trial^24^ (Apalutamide Plus Abiraterone Acetate and Prednisone Versus Placebo Plus Abiraterone and Prednisone in Metastatic, Castration-Resistant Prostate Cancer) and the ELM-PC-5 trial^25^ (Evaluation of the Lyase Inhibitor Orteronel in Metastatic Prostate Cancer 5). By analyzing the association of the BRS with OS and radiographic progression-free survival (rPFS) in these robust datasets, we sought to confirm our preliminary observations and further elucidate the role of this score in providing a reliable assessment of prognosis in patients with mCRPC.

## Methods

### Data Origin

For this prognostic study, we conducted a post hoc analysis using data from the ACIS and ELM-PC-5 phase 3 randomized clinical trials (ClinicalTrials.gov identifiers NCT02257736 and NCT01193257, respectively). Formal ethical approval for this post hoc analysis was waived by the local ethics committee (Medical Faculty of Bonn University) because the study utilized deidentified patient data from previously approved trials. The original trials were conducted in accordance with the ethical principles of the Declaration of Helsinki,^26^ and all participants provided written informed consent prior to their enrollment. Data from the ACIS trial were made accessible by the Yale University Open Data Access (YODA) Project. Data from the ELM-PC-5 trial are available from the Vivli Center for Global Clinical Research Data (Vivli identifier 00009924). As a validation of an existing prognostic model, this study followed the Transparent Reporting of a Multivariable Prediction Model for Individual Prognosis or Diagnosis (TRIPOD) reporting guideline.

### Patient Cohort

The full designs, procedures, and final analyses of both the ACIS and ELM-PC-5 trials have been published previously.^24,25^ In brief, the ACIS trial enrolled 982 male patients (aged ≥18 years) from 167 sites in 17 countries in North America, Europe, the Asia Pacific region, Africa, and South America in a docetaxel-naive and androgen receptor pathway inhibitor (ARPI)–naive first-line mCRPC setting between December 2014 and August 2016.^25^ Patients were randomized to receive either apalutamide plus abiraterone acetate and prednisone or placebo plus abiraterone and prednisone. At final analysis, the median follow-up was 54.8 months (IQR, 51.5-58.4 months). Relevant inclusion criteria in the context of the present work were a hemoglobin level greater than 9 g/dL and an ECOG PS score of 0 to 1. The median age of patients was 71 years in both the apalutamide-abiraterone-prednisone arm (IQR, 66-78 years) and the abiraterone-prednisone arm (IQR, 65-77 years).

In the ELM-PC-5 trial, 1099 male patients (aged ≥18 years) with mCRPC were enrolled from 260 study centers in 42 countries.^24^ They were randomly assigned to receive either orteronel and prednisone or placebo and prednisone subsequent to a progression to docetaxel chemotherapy between October 2010 and February 2013, representing a non–first-line setting. At final analysis, the median follow-up was 10.6 months (range, 0.2-29.5 months) and 10.7 months (range, 0.4-27.1 months) in the orteronel-prednisone and placebo-prednisone groups, respectively. The relevant inclusion criterion was an ECOG PS score of 0 to 2.^23^ The median age of patients was 69.5 years (range, 43-89 years) in the orteronel-prednisone arm and 70 years (range, 48-87 years) in the placebo-prednisone arm.

### Cohort Selection and Rationale

The ACIS and ELM-PC-5 trials were deliberately chosen for this validation study because they represent distinct, well-characterized cohorts across the mCRPC continuum (ACIS: first-line/ARPI-naive setting; and ELM-PC-5: postdocetaxel setting). Both trials were selected primarily because they were negative regarding their key end point of OS. This absence of a significant survival benefit for the investigational arms in the ACIS trial^24^ (median OS, 36.2 [95% CI, 32.8-38.8] months in the apalutamide-abiraterone-prednisone arm vs 33.7 [95% CI, 31.2-38.3] months in the abiraterone-prednisone arm; hazard ratio [HR], 0.95 [95% CI, 0.81-1.11]; *P* = .50) and the ELM-PC-5 trial^25^ (median OS, 17.0 [95% CI, 15.2-19.9] months in the orteronel-prednisone arm vs 15.2 [95% CI, 13.5-16.9] months in the placebo-prednisone arm; HR, 0.89 [95% CI, 0.74-1.01]; *P* = .19) minimizes the risk of introducing a treatment-specific bias into a purely prognostic analysis. Therefore, combining the treatment and placebo or control groups maximizes the statistical power and external validity of the prognostic assessment across a broad base of patients with mCRPC. To further ensure the robustness of our findings, the prognostic capability of the BRS was confirmed in a dedicated sensitivity analysis using only the control group cohorts.

Although they were initially considered for inclusion as outlined in the Vivli and YODA data requests, 3 additional mCRPC trials (ClinicalTrials.gov identifiers NCT01695135, NCT01308567, and NCT00887198) were ultimately omitted. These studies excluded patients possessing at least 1 BRS risk factor—specifically liver metastases (NCT00887198) and a hemoglobin level of less than 10.0 g/dL (all 3 trials)—making their data incompatible with the present analysis.

### Baseline and Treatment Data

Age, pretreatment, body mass index (BMI), ECOG PS score, metastatic patterns, and score on question 3 on the Brief Pain Inventory–Short Form (BPI-SF) were collected alongside laboratory values (PSA and hemoglobin) at the time of enrollment for both trials. The ECOG PS describes a patient’s ability to perform daily activities and is scored as follows: 0, fully active with no restrictions; 1, restricted in physically strenuous activity but ambulatory and capable of light work; 2, restricted in work activity but ambulatory and capable of self-care; 3, capable of limited self-care; 4, completely disabled; and 5, deceased.^23^ After treatment initiation, the biochemical response, delineated as a reduction in PSA by 50% or greater or 90% or greater from baseline (PSA50 and PSA90, respectively) or below a threshold of 0.2 ng/mL (PSA0.2), was collected.

### Bellmunt Risk Score

The BRS was calculated as described previously, with 1 point added for the presence of each of the following risk factors: an ECOG PS score of 1 or greater, a hemoglobin level of less than 10 g/dL, and liver metastasis. This calculation resulted in an integer score potentially ranging from 0 to 3.^18^

### End Points

We collected data for OS, defined as the duration from enrollment into each trial treatment until the occurrence of death or loss of follow-up. rPFS was defined as the time from enrollment until radiographically confirmed progression. Comparisons for each end point were made between patients with a BRS of 0, 1, 2, or 3.

### Statistical Analysis

Descriptive statistics included frequencies and proportions for categorical variables. Medians and ranges were reported for continuously coded variables. Differences were detected using the Mann-Whitney *U* test, the Kruskal-Wallis test, or the χ^2^ test as appropriate. The sensitivity analysis utilized only the patients randomized to the control or placebo arms of both trials: placebo plus abiraterone acetate and prednisone in the ACIS trial and placebo plus prednisone in the ELM-PC-5 trial. A univariable Cox proportional hazards regression model was fitted to assess the association of the BRS with OS. The results were compared with HRs and 95% CIs obtained from the primary combined-cohort analysis. For final survival analysis, a multivariable Cox proportional hazards regression model was fitted to adjust for age, PSA, number of bone metastases (surrogate for metastatic burden), and BPI-SF Pain score (surrogate for symptomatic disease); the Kaplan-Meier method was used to estimate median survival, and concordance indices were calculated. Missing data were handled using listwise deletion, meaning only complete cases were included in the model fit, and no imputation was performed.

Two-sided *P* ≤ .05 was considered statistically significant for all comparisons. All data were coded and analyzed using RStudio, version 2023.09.01+494 (https://CRAN.R-project.org), using the R packages survival, version 3.8-3; rms, version 8.0-0; and finalfit, version 1.1.0. Data were plotted using the package ggpubr, version 0.6.0, in the Vivli Secure Research Environment. Data analyses were conducted between September 2024 and March 2025.

## Results

We identified 678 evaluable male participants in the ACIS trial (median age, 71 years [range, 48-92 years]) and 1078 evaluable male participants in the ELM-PC-5 trial (median age, 70 years [range, 43-89 years]), for a total of 1756 participants, to determine the BRS and its association with survival. The cohorts in this analysis represented a small proportion of the study populations, specifically 8.6% of the ACIS cohort and 16.4% of the ELM-PC-5 cohort. The cohorts predominantly comprised patients of advanced age, with the largest groups in the ACIS cohort being those aged 70 to 74 years (154 [22.7%]) and older than 75 years (227 [33.5%]) (Table 1); in the ELM-PC-5 cohort, the largest groups were aged 66 to 73 years (409 [37.9%]) and 74 to 81 years (285 [26.4%]) (Table 2). In the ACIS cohort, 366 participants (54.0%) presented with a BRS of 0. The remaining scores were distributed as follows: 254 participants (37.5%) had a score of 1, 53 (7.8%) had a score of 2, and 5 (0.7%) had a score of 3. Notably, elevated baseline PSA and higher rates of 10 or more bone lesions or visceral disease were associated with higher scores (Table 1). Within the ELM-PC-5 cohort, 378 participants (35.1%) had a BRS of 0, 523 (48.5%) had a score of 1, 157 (14.6%) had a score of 2, and 20 (1.9%) had a score of 3. Consistent with the ACIS findings, both baseline PSA and the incidence of 10 or more bone lesions or visceral disease were associated with higher BRSs (*P* < .001 each) (Table 2).

**Table 1.** Baseline Characteristics and 1-Year and 2-Year Survival Rates of the ACIS Cohort, Stratified by Bellmunt Risk Score^a^

Characteristic	Overall (n = 678)	Bellmunt Risk Score				*P* value^b^
		0 (n = 366)	1 (n = 254)	2 (n = 53)	3 (n = 5)	
Age group, y						
<65	148 (21.8)	89 (24.3)	51 (20.1)	7 (13.2)	1 (20.0)	.10
65-70	149 (22.0)	85 (23.2)	49 (19.3)	14 (26.4)	1 (20.0)	
70-74	154 (22.7)	85 (23.2)	55 (21.7)	11 (20.8)	3 (60.0)	
>75	227 (33.5)	107 (29.2)	99 (39.0)	21 (39.6)	0	
Treatment arm						
APA plus AA-P	340 (50.1)	193 (52.7)	121 (47.6)	24 (45.3)	2 (40.0)	.51
Placebo plus AA-P	338 (49.9)	173 (47.3)	133 (52.4)	29 (54.7)	3 (60.0)	
PSA, median (range), ng/mL	37.4 (0.5-6050.0)	32.1 (1.4-6050.0)	40.8 (0.5-2780.0)	43.0 (4.0-1350.0)	384 (13.5-1650.0)	.04
No. of bone lesions						
>10	461 (68.0)	277 (75.7)	154 (60.6)	29 (54.7)	1 (20.0)	<.001
≤10	216 (31.9)	89 (24.3)	99 (39.0)	24 (45.3)	4 (80.0)	
Missing	1 (0.1)	0	1 (0.4)	0	0	
Visceral disease						
Absent	536 (79.1)	313 (85.5)	193 (76.0)	28 (52.8)	2 (40.0)	<.001
Present	142 (20.9)	53 (14.5)	61 (24.0)	25 (47.2)	3 (60.0)	
Survival rate (95% CI), %						
1 y	87.2 (84.7-89.8)	92.2 (89.5-95.0)	84.4 (80.0-89.0)	70.1 (59.4-84.4)	25.0 (4.6-100)	<.001
2 y	61.5 (57.9-65.3)	71.4 (66.8-76.3)	52.8 (46.9-59.5)	39.3 (27.9-55.3)	NA	

Abbreviations: AA-P, abiraterone acetate with prednisone; APA, apalutamide; NA, not applicable; PSA, prostate-specific antigen.

^a^ Unless indicated otherwise, values are presented as the No. (%) of participants.

^b^ Calculated using the Mann-Whitney *U* test, Kruskal-Wallis test, χ^2^ test, or log-rank test.

**Table 2.** Baseline Characteristics and 1-Year and 2-Year Survival Rates of the ELM-PC-5 Cohort, Stratified by Bellmunt Risk Score^a^

Characteristic	Overall (n = 1078)	Bellmunt Risk Score				*P* value^b^
		0 (n = 378)	1 (n = 523)	2 (n = 157)	3 (n = 20)	
Age group, y						
50-57	71 (6.6)	29 (7.7)	29 (5.5)	10 (6.4)	3 (15.0)	.07
58-65	250 (23.2)	93 (24.6)	122 (23.3)	31 (19.7)	4 (20.0)	
66-73	409 (37.9)	154 (40.7)	183 (35.0)	65 (41.4)	7 (35.0)	
74-81	285 (26.4)	87 (23.0)	149 (28.5)	44 (28.0)	5 (25.0)	
82-89	52 (4.8)	9 (2.4)	36 (6.9)	6 (0.6)	1 (5.0)	
Missing	11 (1.0)	6 (1.6)	4 (0.8)	1 (0.6)	0	
Treatment arm						
Orteronel plus prednisone	722 (67.0)	260 (68.8)	354 (67.7)	96 (61.1)	12 (60.0)	.32
Placebo plus prednisone	356 (33.0)	118 (31.2)	169 (32.3)	61 (38.9)	8 (40.0)	
PSA, median (range), ng/mL	128 (0.2-19 000.0)	86.8 (1.86- 6050.0)	131 (0.2-19 000.0)	248 (2.7-8460.0)	347 (7.6-2390.0)	<.001
No. of bone lesions						
>10	615 (57.1)	196 (51.9)	224 (42.8)	39 (24.8)	4 (20.0)	<.001
≤10	463 (42.9)	182 (48.1)	299 (57.2)	118 (75.2)	16 (80.0)	
Visceral disease						
Absent	788 (73.1)	318 (84.1)	400 (76.5)	70 (44.6)	0	<.001
Present	290 (26.9)	60 (15.9)	123 (23.5)	87 (55.4)	20 (100)	
No. of prior chemotherapy lines						
1	820 (76.1)	295 (78.0)	404 (77.2)	110 (70.1)	11 (55.0)	.03
≥2	258 (23.9)	83 (22.0)	119 (22.8)	47 (29.9)	9 (45.0)	
Survival rate (95% CI)						
1 y	61.1 (58.0-64.3)	76.0 (71.5-80.7)	60.4 (56.0-65.1)	32.2 (25.2-41.1)	18.0 (6.8-48.0)	<.001
2 y	34.1 (29.8-39.2)	46.5 (37.5-57.6)	31.0 (25.3-37.9)	17.7 (11.1-28.2)	9.0 (1.6-49.1)	

Abbreviation: PSA, prostate-specific antigen.

^a^ Unless indicated otherwise, values are presented as the No. (%) of participants.

^b^ Calculated using the Mann-Whitney *U* test, Kruskal-Wallis test, χ^2^ test, or log-rank test.

### Overall Survival

#### ACIS Trial

**Figure 1.**
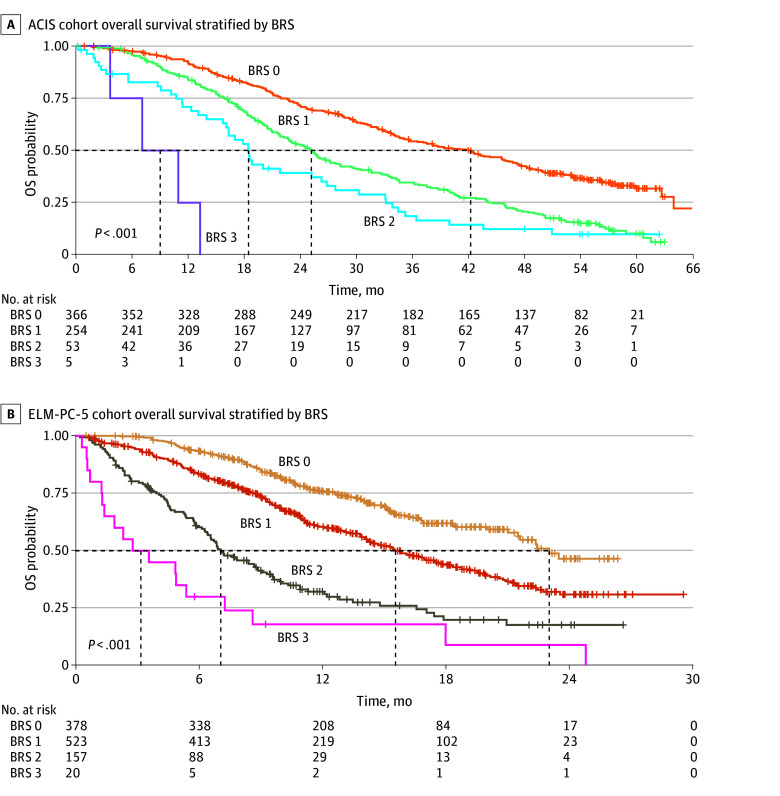
Kaplan-Meier Estimators of Overall Survival (OS) Stratified by Bellmunt Risk Score (BRS) in the ACIS and ELM-PC-5 Cohorts

OS in the ACIS trial was significantly stratified by BRS. For participants with a BRS of 0, the median OS was 42.2 months (95% CI, 35.7-46.7 months). This decreased progressively with increasing BRS, yielding 25.2 months (95% CI, 22.7-28.2 months) for participants with a BRS of 1, 18.5 months (95% CI, 16.1-26.8 months) for those with a BRS of 2, and 9.1 months (95% CI, 3.7 months to not reached) for those with a BRS of 3. The 2-year OS rate was 71.4% (95% CI, 66.8%-76.3%) for participants with a BRS of 0 compared with no participants with a BRS of 3 reaching this landmark (Table 1). Kaplan-Meier analysis confirmed a highly significant difference in OS across risk groups (log-rank *P* < .001) (Figure 1A). The C-index was 0.60 (95% CI, 0.58-0.62).

**Figure 2.**
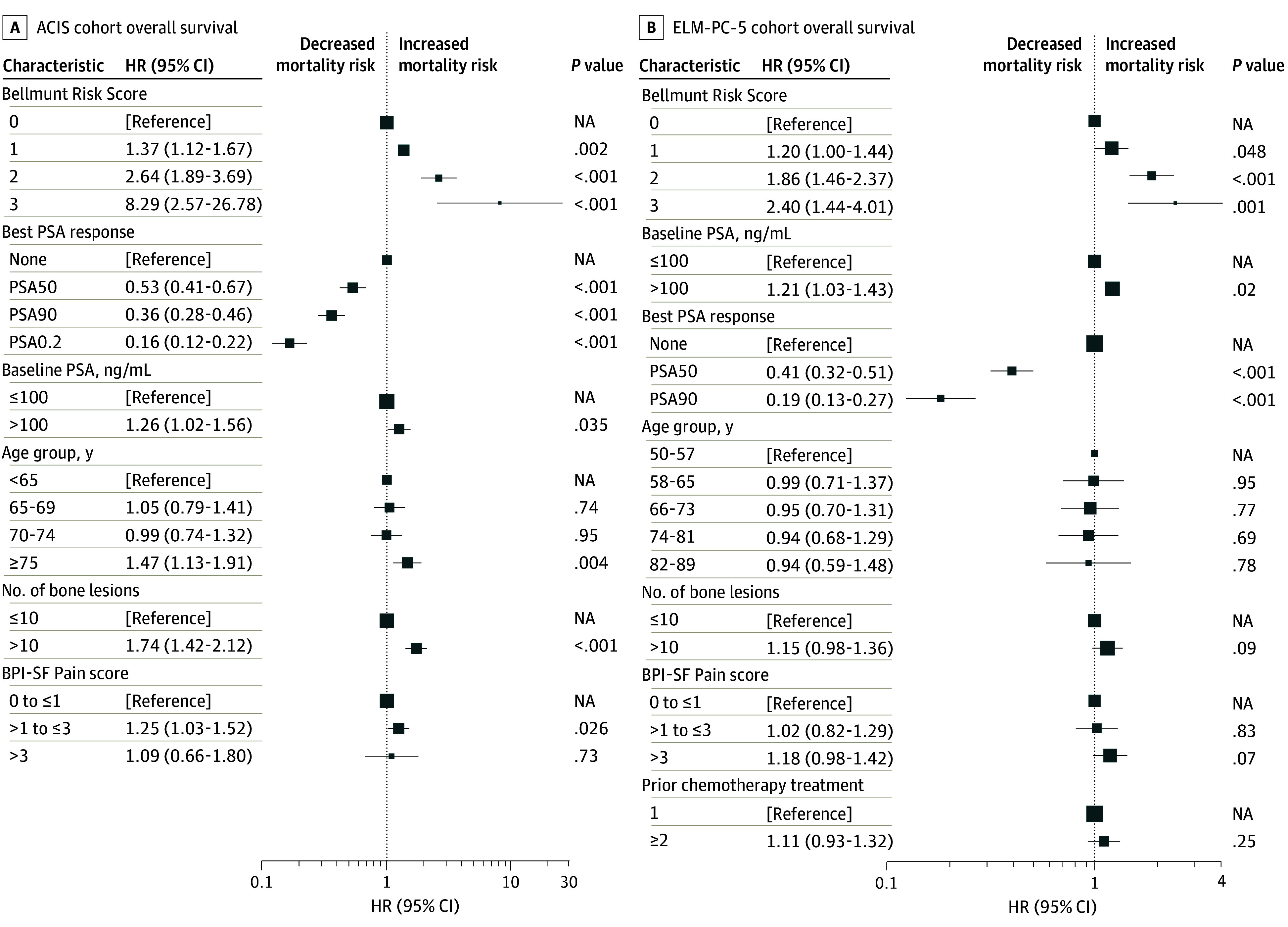
Multivariable Cox Proportional Hazards Regression Analysis of Baseline Variables Alongside Prostate-Specific Antigen (PSA) Response as Predictors of Overall Survival (OS) in the ACIS and ELM-PC-5 Cohorts BPI-SF indicates Brief Pain Inventory–Short Form; HR, hazard ratio; NA, not applicable; PSA0.2, PSA less than the threshold of 0.2 ng/mL; PSA50, PSA reduction by 50% or greater from baseline; PSA90, PSA reduction by 90% or greater from baseline.

In multivariable Cox proportional hazards regression analysis, the BRS remained a strong independent predictor of mortality even when accounting for other significant covariates, including PSA response. Compared with participants with a BRS of 0, adjusted HRs (AHRs) for OS were 1.37 for those with a BRS of 1 (95% CI, 1.12-1.67; *P* = .002), 2.64 for those with a BRS of 2 (95% CI, 1.89-3.69; *P* < .001), and 8.29 for those with a BRS of 3 (95% CI, 2.57-26.78; *P* < .001) (Figure 2A). Other significant covariates in the multivariable model included baseline PSA greater than 100 ng/mL (HR, 1.26 [95% CI, 1.02-1.56]; *P* = .04); achievement of PSA50 (HR, 0.53 [95% CI, 0.41-0.67]), PSA90 (HR, 0.36 [95% CI, 0.28-0.46]), or PSA0.2 (HR, 0.16 [95% CI, 0.12-0.22]) (all *P* < .001); more than 10 bone lesions (HR, 1.74 [95% CI, 1.42-2.12]; *P* < .001); age 75 years or older (HR, 1.47 [95% CI, 1.13-1.91]; *P* = .004); and BPI-SF Pain score of greater than 1 up to 3 (HR, 1.25 [95% CI, 1.03-1.52]; *P* = .03) (eTable 1 in Supplement 1).

#### ELM-PC-5 Trial

In the ELM-PC-5 trial, OS again was associated with BRS (log-rank *P* < .001) (Figure 1B). Individuals with a BRS of 0 exhibited a median OS of 23.0 (95% CI, 21.50 to not reached) months. A consistent decrease in median OS was observed with higher risk scores, with values of 15.6 months (95% CI, 14.3-17.8 months) for those with a BRS of 1, 7.1 months (95% CI, 6.4-9.1 months) for those with a BRS of 2, and 3.2 months (95% CI, 1.4-8.6 months) for those with a BRS of 3. The C-index was 0.65 (95% CI, 0.63-0.68). The 2-year OS rate was 46.5% (95% CI, 37.5%-57.6%) for participants with a BRS of 0 compared with 9.0% (95% CI, 1.7%-49.1%) for those with a BRS of 3 (Table 2).

In multivariable Cox proportional hazards regression analysis, BRS remained a strong independent predictor of mortality (Figure 2B). Compared with participants with a BRS of 0, AHRs for OS were 1.65 for those with a BRS of 1 (95% CI, 1.31-2.07), 2.93 for those with a BRS of 2 (95% CI, 2.22-3.87), and 4.43 for those with a BRS of 3 (95% CI, 2.65-7.40) (all *P* < .001) (eTable 2 in Supplement 1). Other significant covariates in the multivariable model included baseline PSA greater than 100 ng/mL (HR, 1.63 [95% CI, 1.34-1.99]; *P* < .001), more than 10 bone lesions (HR, 1.64 [95% CI, 1.34-2.01]; *P* < .001), BPI-SF Pain score of greater than 3 (HR, 1.45 [95% CI, 1.17-1.81]; *P* = .001), and best PSA response of PSA50 (HR, 0.30 [95% CI, 0.21-0.41]; *P* < .001) and PSA90 (HR, 0.18 [95% CI, 0.10-0.30]; *P* < .001) (eTable 2 in Supplement 1).

### Radiographic Progression-Free Survival

#### ACIS Trial

**Figure 3.**
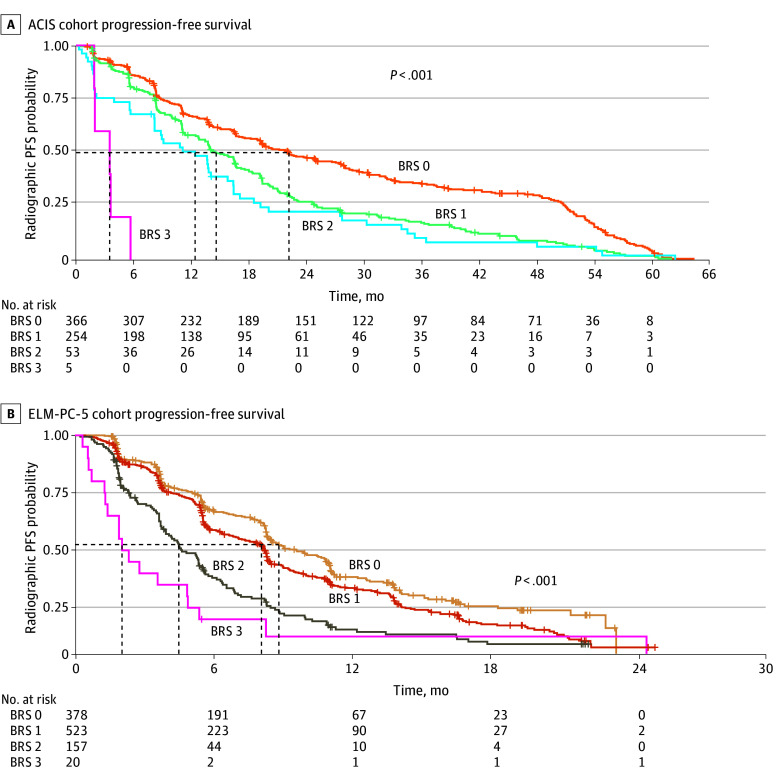
Kaplan-Meier Estimators of Radiographic Progression-Free Survival (PFS) Stratified by Bellmunt Risk Score (BRS) in the ACIS and ELM-PC-5 Cohorts

In the ACIS trial, rPFS was also predicted by BRS. For participants with a BRS of 0, the median rPFS was 22.1 months (95% CI, 19.2-27.6 months). This duration progressively shortened with increasing risk, yielding 14.6 months (95% CI, 13.4-16.7 months) for those with a BRS of 1, 12.4 months (95% CI, 8.2-16.4 months) for those with a BRS of 2, and 3.5 months (95% CI, 1.9 months to not reached) for those with a BRS of 3 (log-rank *P* < .001) (Figure 3A). In multivariable Cox proportional hazards regression analysis, BRS remained an independent predictor of rPFS (eTable 3 in Supplement 1). Compared with participants with a BRS of 0, the AHRs for radiographic progression or death were 1.19 (95% CI, 0.99-1.43; *P* = .07) for those with a BRS of 1, 1.52 (95% CI, 1.11-2.08; *P* = .008) for those with a BRS of 2, and 7.32 (95% CI, 2.64-20.29; *P* < .001) for those with a BRS of 3. The other significant covariate in the multivariable model was the presence of more than 10 bone lesions (HR, 1.53 [95% CI, 1.26-1.86]; *P* < .001). Additionally, achieving a better PSA response was associated with reduced risk, with HRs of 0.48 (95% CI, 0.38-0.61) for PSA50, 0.33 (95% CI, 0.26-0.41) for PSA90, and 0.17 (95% CI, 0.13-0.23) for PSA0.2 (all *P* < .001).

#### ELM-PC-5 Trial

Similarly, in the ELM-PC-5 trial, rPFS exhibited an association with BRS (log-rank *P* < .001) (Figure 3B). Individuals with a BRS of 0 experienced a median rPFS of 8.84 months (95% CI, 8.32-10.82 months). A consistent decrease in median rPFS was observed with higher risk scores, with associated values of 8.09 months (95% CI, 6.67-8.32 months) for those with a BRS of 1, 4.50 months (95% CI, 3.81-5.36 months) for those with a BRS of 2, and 2.04 months (95% CI, 1.41-5.39 months) for those with a BRS of 3.

In multivariable Cox proportional hazards regression analysis, BRS independently predicted radiographic progression or death. Compared with participants with a BRS of 0, AHRs were 1.20 (95% CI, 1.00-1.44; *P* = .05) for those with a BRS of 1, 1.86 (95% CI, 1.46-2.37; *P* < .001) for those with a BRS of 2, and 2.40 (95% CI, 1.44-4.01; *P* = .001) for those with a BRS of 3 (eTable 4 in Supplement 1). The other significant covariate in the multivariable model was baseline PSA greater than 100 ng/mL (HR, 1.21 [95% CI, 1.03-1.43]; *P* = .02). Additionally, achieving a better PSA response was associated with reduced risk, with HRs of 0.41 (95% CI, 0.32-0.51) and 0.19 (95% CI, 0.13-0.27) for PSA50 and PSA90, respectively (both *P* < .001).

The prognostic effect of the BRS was robust. Univariable HRs for OS and rPFS in the placebo-only cohorts showed consistent magnitude and statistical significance compared with the overall study cohorts (eTable 5 in Supplement 1).

## Discussion

Prognostic models play a critical role in guiding clinical decision-making, stratifying patients for treatment, and counseling regarding disease trajectory in advanced prostate cancer.^27,28^ The BRS, leveraging readily available baseline clinical parameters, stands out as a robust and well-validated tool, consistently demonstrating its utility in high-quality prospective data of the ACIS and ELM-PC-5 clinical trials in this study.

The BRS is derived from a limited set of easily obtainable clinical variables and key prognostic domains by integrating ECOG PS score and hemoglobin level as indicators of functional decline, alongside liver metastases and anemia as markers and promotors of tumor aggressiveness.^29,30,31,32,33^ Together, these parameters account for substantial variability in patient outcomes.^10^ The accessibility and ease of interpretation of the BRS enables clear prognostic communication, supports expectation management, and strengthens shared decision-making in patients with mCRPC.

The findings from both the ACIS and ELM-PC-5 trials consistently underscore the utility of the BRS as a powerful prognostic indicator in mCRPC survival. Across both cohorts, patients with a higher BRS demonstrated a clear and consistently poorer outlook in terms of both OS and rPFS. These consistent differentiations across distinct patient populations strongly validate the previously observed ability of the BRS to discriminate risk across different treatment lines in mCRPC.^20^ Furthermore, the fundamental prognostic factors captured by the BRS (functional status, anemia, and visceral metastasis burden) appear robust regardless of modern treatment modality. Indeed, in a 2025 study, the utility of the BRS as a predictor of OS was confirmed in a cohort of patients receiving radioligand therapy, demonstrating its enduring value even with integration of novel, targeted agents.^34^ Adding to the BRS’s consistency across different mCRPC treatment lines, recent clinical data suggest potential applicability in metastatic hormone-sensitive prostate cancer (mHSPC).^35^

To rigorously quantify the observed prognostic value independent of confounding factors, we performed multivariable Cox proportional hazards regression analyses for both trials. Continuous variables such as age were modeled using clinically defined, factor-coded groups due to the deidentified structure of the available underlying data. Despite this constraint, the BRS was confirmed as an independent predictor of OS and rPFS. Although PSA response was also associated with improved prognosis, the BRS offers critical baseline prognostic value—independent of treatment response requiring follow-up data—enabling early risk stratification before therapy initiation. Additionally, especially in later-line settings as demonstrated by the ELM-PC-5 trial, only a minority of patients achieved a PSA50 or PSA90 response,^36,37^ thereby diminishing the overall utility of PSA dynamics as a sole prognostic marker. Consequently, the need for robust baseline prognostic tools becomes even more critical.

### Strengths and Limitations

Our findings reinforce the utility of the BRS irrespective of PSA response and as a valuable baseline predictor even in pretreated mCRPC populations. However, we recognize that this post hoc analysis was limited by the data collected in the original trials. Crucially, factors such as genetic germline testing, family history, and detailed racial and ethnic demographics were either not systematically assessed or were removed during the deidentification process, precluding their incorporation into our multivariable models. These omissions limit the study’s ability to assess the BRS across diverse genetic, molecular, and racial and ethnic risk groups.

Even without these covariates, the BRS effectively stratified risk, most notably at the upper extremes where a score of 2 and 3 identified the most vulnerable patients. The cohort in this analysis represented a small proportion of the study populations, specifically 8.6% of the ACIS cohort and 16.4% of the ELM-PC-5 cohort. Consequently, the BRS identifies a particularly vulnerable subgroup with a critical need for enhanced palliative care and the development of novel therapeutic strategies. It is important to acknowledge that the 95% CIs for the subgroups of patients with a BRS of 3 in both trials were notably wide due to this small sample size, indicating statistical imprecision. Nevertheless, the clinical trials included in this analysis were subject to selection bias due to narrow inclusion criteria (eg, ECOG PS score of 0-1 and hemoglobin >9.0 g/dL in the ACIS trial).^38^ This may limit the generalizability of these proportions to a broader clinical mCRPC population. In contrast to our trial cohorts, clinical data from patients with mCRPC showed a substantially higher prevalence of BRSs of 2 or greater, with 27.2% reported in the first-line setting,^20^ 30.1% in later lines,^34^ and 20.1% even in the prior hormone-sensitive stage of the disease (mHSPC).^35^ This finding suggests that an increased overall prevalence of high-risk patients might be observed in routine clinical settings, while calling for further external validation.

This difference in cohort composition likely contributes to the variation observed in prognostic accuracy metrics.^39^ The C-indices reported (0.60 for ACIS and 0.65 for ELM-PC-5) contrast with those previously reported from clinical mCRPC cohorts (ranging from 0.66-0.72),^20,34^ likely due to the substantially higher proportion of patients at low risk (BRS of 0) in these clinical trial populations. When comparing these metrics with complex prognostic models such as the Halabi and Armstrong nomograms (C-indices of 0.70-0.74),^40^ it must be noted that unlike these, the BRS is an ordinal, categorical score. This structure inherently leads to frequent prediction ties, which mathematically depresses the calculated C-index value and complicates direct comparisons with continuous models. Although a C-index of 0.5 represents random chance and 0.7 is often cited as a benchmark for good discrimination, such thresholds are increasingly criticized as arbitrary, because they are highly dependent on underlying cohort characteristics.^39^ This is reflected by the improved metrics in the ELM-PC-5 trial, in which higher BRS values were more frequent than in the ACIS cohort. Despite these constraints, our findings confirm that the BRS delivers clinically meaningful and pragmatic prognostic utility using only a fraction of the variables required by established, more complex models.

## Conclusions

Collectively, the findings of this prognostic study suggest that the BRS can effectively aid clinical decision-making across treatment lines in mCRPC. In light of the rising incidence of metastatic prostate cancer, the ability of the BRS to categorize patients into distinct risk groups at baseline allows for more informed discussions about prognosis, helps guide therapeutic choices (eg, considering intensity of treatment or participation in clinical trials), and can be integrated into future clinical trial designs for patient stratification.^41^ The sustained prognostic power of the BRS, especially its independence from dynamic markers such as PSA response in heavily pretreated populations, reinforces its valuable role in supporting treatment of patients with mCRPC.

## Notes

Supplement 1. **eTable 1.** Univariable and Multivariable Cox Proportional Hazards Regression Analysis for Predictors of Overall Survival in the ACIS Trial **eTable 2.** Multivariable Cox Proportional Hazards Regression Analysis for Predictors of Overall Survival in the ELM-PC-5 Trial **eTable 3.** Multivariable Cox Proportional Hazards Regression Analysis for Predictors of Radiographic Progression-Free Survival in the ACIS Trial **eTable 4.** Multivariable Cox Proportional Hazards Regression Analysis for Predictors of Radiographic Progression-Free Survival in the ELM-PC-5 Trial **eTable 5.** Hazard Ratios for Overall Survival (OS) and Progression-Free Survival (PFS) Compared Between All Patients and Only Placebo-Treated Patients by Univariable Cox Proportional Hazards Regression Analysis

Supplement 2.
Data Sharing Statement
