# Supplementary material for: Bellmunt Risk Score as a Prognostic Tool in Metastatic Castration-Resistant Prostate Cancer Survival
Source: JAMA Netw Open. 2026 Mar 6;9(3):e260300. doi: 10.1001/jamanetworkopen.2026.0300 (PMC12966927; doi:10.1001/jamanetworkopen.2026.0300)
Supplement: Supplement 1. — eTable 1. Univariable and Multivariable Cox Proportional Hazards Regression Analysis for Predictors of Overall Survival in the ACIS Trial eTable 2. Multivariable Cox Proportional Hazards Regression Analysis for Predictors of Overall Survival in the ELM-PC-5 Trial eTable 3. Multivariable Cox Proportional Hazards Regression Analysis for Predictors of Radiographic Progression-Free Survival in the ACIS Trial eTable 4. Multivariable Cox Proportional Hazards Regression Analysis for Predictors of Radiographic Progression-Free Survival in the ELM-PC-5 Trial eTable 5. Hazard Ratios for Overall Survival (OS) and Progression-Free Survival (PFS) Compared Between All Patients and Only Placebo-Treated Patients by Univariable Cox Proportional Hazards Regression Analysis [file jamanetwopen-e260300-s001.pdf]

Supplemental Online Content

Büttner T, Klümper N, Ellinger J, Ritter M, Krausewitz P. Bellmunt Risk Score as a prognostic tool in metastatic castration-resistant prostate cancer survival. *JAMA Netw Open*. 2026;9(3):e260300. doi:10.1001/jamanetworkopen.2026.0300

eTable 1. Univariable and Multivariable Cox Proportional Hazards Regression Analysis for Predictors of Overall Survival in the ACIS Trial

eTable 2. Multivariable Cox Proportional Hazards Regression Analysis for Predictors of Overall Survival in the ELM-PC-5 Trial

eTable 3. Multivariable Cox Proportional Hazards Regression Analysis for Predictors of Radiographic Progression-Free Survival in the ACIS Trial

eTable 4. Multivariable Cox Proportional Hazards Regression Analysis for Predictors of Radiographic Progression-Free Survival in the ELM-PC-5 Trial

eTable 5. Hazard Ratios for Overall Survival (OS) and Progression-Free Survival (PFS) Compared Between All Patients and Only Placebo-Treated Patients by Univariable Cox Proportional Hazards Regression Analysis

This supplemental material has been provided by the authors to give readers additional information about their work.

© 2026 Büttner T et al. *JAMA Network Open*.

eTable 1. Univariable and Multivariable Cox Proportional Hazards Regression Analysis for Predictors of Overall Survival in the ACIS Trial

**Parameter**	**Group**	**N=**	**HR for OS (univariable)**	**HR for OS (multivariable)**
Bellmunt Risk Score	0	366 (54.0)	-	-
	1	254 (37.5)	1.90 (1.57-2.30, p<0.001)	1.37 (1.12-1.67, p=0.002)
	2	53 (7.8)	2.73 (1.98-3.77, p<0.001)	2.64 (1.89-3.69, p<0.001)
	3	5 (0.7)	16.08 (5.86-44.12, p<0.001)	8.29 (2.57-26.78, p<0.001)
Best PSA Response	None	152 (22.4)	-	-
	PSA50	197 (29.1)	0.52 (0.41-0.65, p<0.001)	0.53 (0.41-0.67, p<0.001)
	PSA90	189 (27.9)	0.36 (0.28-0.46, p<0.001)	0.36 (0.28-0.46, p<0.001)
	PSA0.2	140 (20.6)	0.14 (0.10-0.19, p<0.001)	0.16 (0.12-0.22, p<0.001)
Baseline PSA (ng/mL)	<100	500 (73.7)	-	-
	>100	178 (26.3)	1.72 (1.41-2.10, p<0.001)	1.26 (1.02-1.56, p=0.04)
Age (years)	<65	148 (21.8)	-	-
	65-69	149 (22.0)	1.18 (0.90-1.56, p=0.24)	1.05 (0.79-1.41, p=0.74)
	70-74	154 (22.7)	1.13 (0.86-1.50, p=0.38)	0.99 (0.74-1.32, p=0.95)
	>=75	227 (33.5)	1.55 (1.20-2.00, p=0.001)	1.47 (1.13-1.91, p=0.004)
Number of Bone Lesions	<=10	461 (68.1)	-	-
	>10	216 (31.9)	2.33 (1.93-2.82, p<0.001)	1.74 (1.42-2.12, p<0.001)
BPI-SF Pain score	0 to <=1	424 (63.5)	-	-
	>1 to <=3	218 (32.6)	1.51 (1.25-1.83, p<0.001)	1.25 (1.03-1.52, p=0.03)
	>3	26 (3.9)	1.12 (0.68-1.82, p=0.66)	1.09 (0.66-1.80, p=0.73)
HR = Hazard Ratio, OS = overall survival, BPI-SF = Brief Pain Inventory Short Form question 3				

© 2026 Büttner T et al. *JAMA Network Open*.

eTable 2. Multivariable Cox Proportional Hazards Regression Analysis for Predictors of Overall Survival in the ELM-PC-5 Trial

Parameter	Group	n= (%)	HR for OS (univariable)	HR for OS (multivariable)
Bellmunt Risk Score	0	378 (35.1)	-	-
	1	523 (48.5)	1.76 (1.42-2.19, p<0.001)	1.65 (1.31-2.07, p<0.001)
	2	157 (14.6)	4.01 (3.09-5.20, p<0.001)	2.93 (2.22-3.87, p<0.001)
	3	20 (1.9)	8.35 (5.08-13.73, p<0.001)	4.43 (2.65-7.40, p<0.001)
Baseline PSA (ng/mL)	<100	482 (44.7)	-	-
	>100	596 (55.3)	2.03 (1.69-2.45, p<0.001)	1.63 (1.34-1.99, p<0.001)
Best PSA Response	None	829 (76.9)	-	-
	PSA50	161 (14.9)	0.31 (0.23-0.43, p<0.001)	0.30 (0.21-0.41, p<0.001)
	PSA90	88 (8.2)	0.18 (0.11-0.31, p<0.001)	0.18 (0.10-0.30, p<0.001)
Age (years)	50-57	71 (6.7)	-	-
	58-65	250 (23.4)	0.97 (0.64-1.45, p=0.86)	1.10 (0.73-1.66, p=0.66)
	66-73	409 (38.3)	1.09 (0.74-1.60, p=0.66)	1.28 (0.87-1.88, p=0.22)
	74-81	285 (26.7)	1.29 (0.87-1.92, p=0.20)	1.34 (0.90-2.00, p=0.15)
	82-89	52 (4.9)	1.24 (0.73-2.12, p=0.43)	1.27 (0.73-2.21, p=0.40)
Number of Bone Lesions	<=10	463 (42.9)	-	-
	>10	615 (57.1)	2.11 (1.75-2.55, p<0.001)	1.64 (1.34-2.01, p<0.001)
BPI-SF Pain Score	0 to <=1	373 (34.9)	-	-
	>1 to <=3	198 (18.5)	1.32 (1.01-1.71, p=0.04)	1.21 (0.92-1.58, p=0.17)
	>3	499 (46.6)	1.82 (1.48-2.24, p<0.001)	1.45 (1.17-1.81, p=0.001)
Prior Chemotherapy	1	820 (76.1)	-	-
	2 or more	258 (23.9)	1.24 (1.01-1.52, p=0.04)	1.01 (0.82-1.24, p=0.94)
HR = Hazard Ratio, OS = overall survival, BPI-SF = Brief Pain Inventory Short Form question 3				

© 2026 Büttner T et al. *JAMA Network Open*.

eTable 3. Multivariable Cox Proportional Hazards Regression Analysis for Predictors of Radiographic Progression-Free Survival in the ACIS Trial

**Parameter**	**Group**	**N=**	**HR for rPFS (univariable)**	**HR for rPFS (multivariable)**
Bellmunt Risk Score	0	366 (54.0)	-	-
	1	254 (37.5)	1.59 (1.33-1.89, p<0.001)	1.19 (0.99-1.43, p=0.07)
	2	53 (7.8)	1.87 (1.39-2.53, p<0.001)	1.52 (1.11-2.08, p=0.008)
	3	5 (0.7)	12.36 (4.99-30.60, p<0.001)	7.32 (2.64-20.29, p<0.001)
Best PSA Response	No	152 (22.4)	-	-
	PSA50	197 (29.1)	0.46 (0.37-0.58, p<0.001)	0.48 (0.38-0.61, p<0.001)
	PSA90	189 (27.9)	0.31 (0.25-0.39, p<0.001)	0.33 (0.26-0.41, p<0.001)
	PSA0.2	140 (20.6)	0.15 (0.11-0.20, p<0.001)	0.17 (0.13-0.23, p<0.001)
Baseline PSA (ng/mL)	<100	500 (73.7)	-	-
	>100	178 (26.3)	1.53 (1.28-1.84, p<0.001)	1.07 (0.88-1.31, p=0.48)
Age (years)	<65	148 (21.8)	-	-
	65-69	149 (22.0)	1.13 (0.88-1.46, p=0.34)	1.09 (0.83-1.41, p=0.54)
	70-74	154 (22.7)	1.06 (0.82-1.36, p=0.66)	0.89 (0.68-1.15, p=0.36)
	>=75	227 (33.5)	1.26 (1.00-1.58, p=0.05)	1.20 (0.95-1.52, p=0.14)
Number of Bone Lesions	<=10	461 (68.1)	-	-
	>10	216 (31.9)	2.02 (1.69-2.41, p<0.001)	1.53 (1.26-1.86, p<0.001)
BPI-SF Pain score	0 to <=1	424 (63.5)	-	-
	>1 to <=3	218 (32.6)	1.21 (1.02-1.44, p=0.03)	1.04 (0.87-1.25, p=0.64)
	>3	26 (3.9)	1.18 (0.74-1.88, p=0.48)	1.31 (0.82-2.11, p=0.26)

HR = Hazard Ratio, rPFS = radiographic progression-free survival, BPI-SF = Brief Pain Inventory Short Form question 3

© 2026 Büttner T et al. *JAMA Network Open*.

eTable 4. Multivariable Cox Proportional Hazards Regression Analysis for Predictors of Radiographic Progression-Free Survival in the ELM-PC-5 Trial

**Parameter**	**Group**	**n= (%)**	**HR for rPFS (univariable)**	**HR for rPFS (multivariable)**
Bellmunt Risk Score	0	378 (35.1)	-	-
	1	523 (48.5)	1.27 (1.08-1.51, p=0.005)	1.20 (1.00-1.44, p=0.05)
	2	157 (14.6)	2.25 (1.80-2.81, p<0.001)	1.86 (1.46-2.37, p<0.001)
	3	20 (1.9)	3.48 (2.13-5.71, p<0.001)	2.40 (1.44-4.01, p=0.001)
Baseline PSA (ng/mL)	<100	482 (44.7)	-	-
	>100	596 (55.3)	1.36 (1.17-1.58, p<0.001)	1.21 (1.03-1.43, p=0.021)
Best PSA Response	None	829 (76.9)	-	-
	PSA50	161 (14.9)	0.40 (0.32-0.50, p<0.001)	0.41 (0.32-0.51, p<0.001)
	PSA90	88 (8.2)	0.18 (0.13-0.27, p<0.001)	0.19 (0.13-0.27, p<0.001)
Age (years)	50-57	71 (6.7)	-	-
	58-65	250 (23.4)	1.06 (0.77-1.47, p=0.71)	0.99 (0.71-1.37, p=0.95)
	66-73	409 (38.3)	0.98 (0.72-1.34, p=0.90)	0.95 (0.70-1.31, p=0.77)
	74-81	285 (26.7)	1.02 (0.74-1.40, p=0.90)	0.94 (0.68-1.29, p=0.69)
	82-89	52 (4.9)	1.03 (0.66-1.62, p=0.88)	0.94 (0.59-1.48, p=0.78)
Number of Bone Lesions	<=10	463 (42.9)	-	-
	>10	615 (57.1)	1.38 (1.19-1.61, p<0.001)	1.15 (0.98-1.36, p=0.10)
BPI-SF Pain Score	0 to <=1	373 (34.9)	-	-
	>1 to <=3	198 (18.5)	1.03 (0.83-1.28, p=0.77)	1.02 (0.82-1.29, p=0.83)
	>3	499 (46.6)	1.37 (1.16-1.62, p<0.001)	1.18 (0.98-1.42, p=0.07)
Prior Chemotherapy	1	820 (76.1)	-	-
	2 or more	258 (23.9)	1.30 (1.09-1.54, p=0.003)	1.11 (0.93-1.32, p=0.25)
HR = Hazard Ratio, rPFS = radiographic progression-free survival, BPI-SF = Brief Pain Inventory Short Form question 3				

© 2026 Büttner T et al. *JAMA Network Open*.

eTable 5. Hazard Ratios for Overall Survival (OS) and Progression-Free Survival (PFS) Compared Between All Patients and Only Placebo-Treated Patients by Univariable Cox Proportional Hazards Regression Analysis

	ACIS		ELM-PC-5	
	**HR for OS (univariable)**	**HR for rPFS (univariable)**	**HR for OS (univariable)**	**HR for rPFS (univariable)**
**Overall cohort**				
BRS=1	1.90 (1.57-2.30, p<0.001)	1.59 (1.33-1.89, p<0.001)	1.76 (1.42-2.19, p<0.001)	1.27 (1.08-1.51, p=0.005)
BRS=2	2.73 (1.98-3.77, p<0.001)	1.87 (1.39-2.53, p<0.001)	4.01 (3.09-5.20, p<0.001)	2.25 (1.80-2.81, p<0.001)
BRS=3	16.08 (5.86-44.12, p<0.001)	12.36 (4.99-30.60, p<0.001)	8.35 (5.08-13.73, p<0.001)	3.48 (2.13-5.71, p<0.001)
**Placebo arm only**				
BRS=1	2.16 (1.64-2.83, p<0.001)	1.78 (1.38-2.30, p<0.001)	2.17 (1.81-4.05, p<0.001)	1.33 (1.00-1.78, p=0.05)
BRS=2	2.64 (1.69-4.13, p<0.001)	1.70 (1.12-2.57, p=0.01)	5.76 (3.63-9.14, p<0.001)	2.15 (1.50-3.08, p<0.001)
BRS=3	13.37 (3.31-56.92, p<0.001)	11.06 (3.42-35.78, p<0.001)	42.33 (18.87-84.99, p<0.001)	14.44 (6.70-31.11, p<0.001)

BRS calculated only from patients in the control arm show similar HRs to the overall cohort.

© 2026 Büttner T et al. *JAMA Network Open*.
