# Supplementary material for: Bellmunt Risk Score as a Prognostic Tool in Metastatic Castration-Resistant Prostate Cancer Survival
Source: JAMA Netw Open. 2026 Mar 6;9(3):e260300. doi: 10.1001/jamanetworkopen.2026.0300 (PMC12966927; doi:10.1001/jamanetworkopen.2026.0300)
Supplement: Supplement 2. — Data Sharing Statement [file jamanetwopen-e260300-s002.pdf]

Data Sharing Statement

Büttner. Bellmunt Risk Score as a Prognostic Tool in Metastatic Castration-Resistant Prostate Cancer Survival. *JAMA Netw Open*. Published March 03, 2026.
doi:10.1001/jamanetworkopen.2026.0300

Data
Data available: No

Additional Information
Explanation for why data not available: The data analyzed in this work is already available at Vivli and the YODA project, see the acknowledgements
